# The Relationship between Systolic Blood Pressure with Anxiety and Depression in Family Caregiver of Hemodialysis Patients at Soehadi Prijonegoro Regional Public Hospital: A Cross-Sectional Study

**DOI:** 10.1192/j.eurpsy.2023.1441

**Published:** 2023-07-19

**Authors:** A. Z. Osman, E. A. P. Susanti, R. I. Estiko, A. D. Widyadhana

**Affiliations:** 1 Department of Psychiatry; 2 Faculty of Medicine, Universitas Islam Indonesia, Yogyakarta, Indonesia

## Abstract

**Introduction:**

The global toll of chronic kidney disease (CKD) is significantly rising and unevenly distributed. In Indonesia, CKD is primarily managed by hemodialysis (HD) because limited resources rule out the possibility of renal transplantation. HD patients are commonly accompanied by caregivers but most studies show neglected the physical and mental health of caregivers.

**Objectives:**

This work aims to know the relationship between anxiety and depression with systolic blood pressure (SBP) in HD caregivers at Soehadi Prijonegoro Regional Public Hospital.

**Methods:**

A cross-sectional study design was conducted to assess the population. This research took place in Soehadi Prijonegoro Regional Public Hospital Sragen Indonesia, at the Hemodialysis department in November 2022, with 31 participants. We assessed their SBP using a sphygmomanometer, and then we interviewed the caregivers using Hamilton Depression Rating Scale (HAM-D or HDRS) and Hamilton Anxiety Rating Scale (HAM-A).

**Results:**

We found that 38,8% of caregivers have hypertension with SBP above 140 mmHg. Around 93.5% and 6.5% of caregivers were found to be mild and mild-moderate anxious. Also, 22.6% were found to have mild depression, while the rest showed the normal result. There is a relationship between SBP and anxiety (p=0.037), while depression is not (p=0.302). However, there is a strong relationship between anxiety and depression (p<0.05), with a correlation coefficient of 0.69.

**Conclusions:**

One-third of the caregivers were found to have hypertension, which is significantly related to anxiety. Furthermore, depression could occur in a patient with anxiety. Thus, caregivers need to maintain their physical and mental health.

**Disclosure of Interest:**

None Declared

